# Recurrent *Lactobacillus* Bacteremia in a Patient With Leukemia

**DOI:** 10.1177/2324709617744233

**Published:** 2017-11-24

**Authors:** Paurush Ambesh, Sarah Stroud, Eva Franzova, Joseph Gotesman, Kavita Sharma, Lawrence Wolf, Stephan Kamholz

**Affiliations:** 1 Maimonides Medical Center, Brooklyn, NY, USA

**Keywords:** *Lactobacillus* bacteremia, leukemia

## Abstract

*Lactobacillus* species are a commensal flora of the human gastrointestinal and the female genitourinary tract. *Lactobacilli* especially the *rhamnosus* species, are common components of commercial probiotics. They are rarely associated with pathology in immunocompetent people, but they have been known to cause dental caries, bacteremia, and endocarditis in patients with suppressed immune function. Cases of *Lactobacillus* bacteremia have been reported in patients with acute myeloid leukemia, large granular lymphocytic leukemia, and in transplant recipients. In this article, we report a strange case of recurrent *Lactobacillus* bacteremia causing multiple episodes of fever of unknown origin in a patient with leukemia. This report is unique as *Lactobacillus* is not recognized as a common source of bacteremia. Moreover, the source of the *bacillus* continued to elude us even after extensive investigation.

## Introduction

Where there’s smoke, there’s fire

—Ancient proverb

*Lactobacillus* species are a commensal flora of the human gastrointestinal and the female genitourinary tract.^1^ *Lactobacilli* especially the *rhamnosus* species, are common components of commercial probiotics.^1-4^ They are rarely associated with pathology in immunocompetent people, but they have been known to cause dental caries, bacteremia, and endocarditis in patients with suppressed immune function.^5^ Cases of *Lactobacillus* bacteremia have been reported in patients with acute myeloid leukemia, large granular lymphocytic leukemia, and in transplant recipients.^6-10^

## Case

An 84-year-old male with past medical history of diabetes mellitus, hyperlipidemia, hypertension, and severe aortic stenosis was admitted for progressive weakness and failure to thrive. He reported a gradual increase in weakness over 4 months that culminated in 2 days of an inability to leave his bed. He denied shortness of breath, night sweats, fever, chest pain, dyspnea on exertion, abdominal pain, nausea, vomiting, or diarrhea. He also denied ever using a probiotic. On physical examination, he was found to have axillary lymphadenopathy bilaterally. Computed tomography (CT) of chest confirmed this lymphadenopathy, and CT of abdomen/pelvis found splenomegaly with additional external iliac, retroperitoneal, and mesenteric lymphadenopathy. However, no possible portal of infection from the gastrointestinal tract to the bloodstream was identified. The patient was subsequently diagnosed with chronic lymphocytic leukemia. Laboratory values showed anemia and thrombocytopenia with a white blood cell count of 10 500. The patient developed fever during the admission and blood cultures were sent. Three sets of blood cultures grew *Lactobacilli*, with *casei* and *paracasei* species subtypes. Urine cultures showed no growth. Since the patient had some dental pain, it was suspected that the source of infection could be in the mouth. Dentistry was consulted, and they performed multiple dental extractions. However, head CT showed no obvious oral source of infection. A transesophageal echocardiogram was done and was negative for endocardial involvement. Even in the absence of a clear source of infection, he was treated with ampicillin-sulbactam for 2 weeks, and subsequent blood cultures showed no growth. He was discharged home after being presumed free of infection.

Two months later, the patient presented in the emergency room with fever, altered mental status, and weakness to the point of being bed-bound. Blood cultures again grew *Lactobacillus rhamnosus.* Blood samples were sent to a specialized center for confirmation of species.

MALDI-TOF (matrix-assisted laser desorption/ionization time-of-flight) technology was utilized for accurate identification of the *Bacillus*.

During the hospital stay, the patient’s type 2 diabetes was managed with carbohydrate-consistent diet and a correction sliding scale insulin regimen. His blood glucose measurements were generally within the 90 to 130 mg/dL range. Hypertension was controlled with a oral β-blocker. Hyperlipidemia was managed with high-intensity statin. For his critical aortic stenosis, cardiology was consulted. However, he had too high a risk even for a transcatheter aortic valve replacement procedure. The lymphoma was in complete remission and did not require further management during this hospitalization.

**Figure 1.**
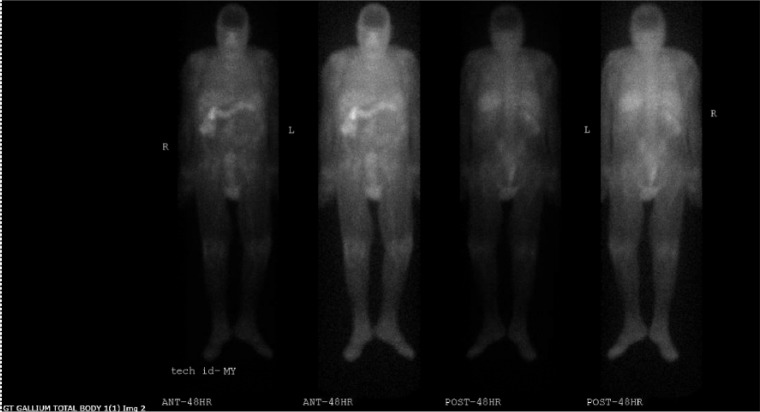
Nuclear medicine gallium scan did not localize any focus of infection.

A full body nuclear medicine gallium scan was performed in the hope of localizing the source of infection. However, the scan showed no focal finding. Mild enhancement in sacrum and coccyx was read by the radiologist as an artifact.[Figure 1] There was no clinical correlation in the patient.

Ampicillin was continued and the patient responded clinically without a new spike of fever. After extensive consultation with the infectious disease department, he was discharged on 4 weeks of ampicillin-sulbactam and gentamicin therapy.

After completion of this therapy, he was put on lifelong oral amoxicillin-clavulanate therapy.

It has been 6 months, and so far he has not presented with infection. He continues to be on prophylactic antibiotic therapy for the remainder of his life.

## Discussion

Immunosuppression has been reported to be a risk factor for *Lactobacillus* bacteremia, along with intravenous catheters, prior hospitalization or surgery, and broad-spectrum antibiotic use.^3,5,11,12^ Another population that seems to be at increased risk are patients with loss of integrity of the intestinal mucosal barrier; several occurrences of *Lactobacillus* bacteremia have been reported in patients with ulcerative colitis.^4,13,14^

The fact that many patients in the inflammatory bowel disease population are concurrently taking immunomodulatory medications cannot be ignored as a contributing risk factor; however, an intriguing case of *Lactobacillus* septicemia in an immunocompetent middle-aged male with ischemic colitis suggests that intestinal pathology alone is sufficient.^15^

*Lactobacillus casei* and its subspecies *rhamnosus* have consistently been the most commonly identified variants in blood cultures.^5,16^ Both organisms have been reported to be resistant to metronidazole and vancomycin, with some strains of *Lactobacillus rhamnosus* known to exhibit multidrug resistance.^1,13,17-19^ Susceptibility to erythromycin, imipenem, and clindamycin have been reported consistently, with ampicillin resistance varying by culture.^8,16,18,20^ Early and appropriate treatment seems essential for decreasing mortality.^3^

*Lactobacillus* species are increasingly being recognized as a source of bacteremia in immunocompromised individuals. They have been previously associated with broad-spectrum antibiotic use, prior hospitalization, and intestinal pathology. Infections due to *Lactobacillus* species have the potential to be fatal and provide a challenge for clinicians given varying antibiotic susceptibilities.^3^

We have reported on a patient with no known probiotic use and no diagnosed intestinal pathology who exhibited recurrent episodes of *Lactobacillus* bacteremia associated with fever and altered mental status. No discrete portal into the bloodstream was ever identified in this patient. The *Lactobacilli* isolated for this patient were susceptible to penicillin and gentamicin. He was thus repeatedly treated with ampicillin-sulbactam and gentamicin. Despite intermittent defervescence and negative blood cultures after treatment, successful eradication of the underlying source is doubtful given his recurrent bacteremia. He continues to be on lifelong oral antibiotic prophylaxis. This approach was finally decided upon, after consultation with infectious disease experts from other tertiary care institutions.

We hope to call attention to the lack of comprehensive data on *Lactobacillus* infections in the literature and to highlight the importance of maintaining a high index of suspicion for *Lactobacillus* infection in immunocompromised patients. A more thorough understanding of possible portals to the bloodstream as well as antibiotic susceptibilities is warranted.
